# Application of biomaterials for the repair and treatment of osteonecrosis of the femoral head

**DOI:** 10.1093/rb/rbz048

**Published:** 2020-01-14

**Authors:** Dewei Zhao, Zhijie Ma

**Affiliations:** Department of Orthopedics, Affiliated Zhongshan Hospital of Dalian University, Liaoning, Dalian 116001, China

**Keywords:** osteonecrosis, femoral head, repair, biomaterials

## Abstract

Osteonecrosis of the femoral head (ONFH) is one of the most common causes of hip disability in young adults. However, its cause and pathogenesis remain unclear, and might be caused by a variety of factors. ONFH mainly occurs in young adults. If not treated, 70–80% of patients would progress into femoral head collapse in 3 years, and eventually require hip arthroplasty. Since these patients are younger and more physically active, multiple revision hip arthroplasty might be needed in their life. Repeated revision hip arthroplasty is difficult and risky, and has many complications, which inevitably affects the physical and mental health of patients. To delay the time of total hip arthroplasty for young adult patients with ONFH, biomaterials are used for its repair, which has a high clinical and social value for the retention of the patient’s own hip function. At present, there are many types of biomaterials used in repairing the femoral head, there is no uniform standard of use and the clinical effects are different. In this review, the main biomaterials used in the repair of ONFH are summarized and analyzed, and the prospects are also described.

## Introduction

Osteonecrosis of the femoral head (ONFH), also known as avascular necrosis (AVN) of the femoral head, was first described in 1738 by Munro. ONFH refers to the death of some osteocytes or the necrosis of some marrow elements caused by venous congestion, impairment or interruption of arterial blood, and subsequent repair, which in turn causes the necrosis of bone tissues. In most cases, these changes ultimately lead to structural changes and the collapse of the femoral head and cause pain and dysfunction of the hip joint [1]. Due to the complex aetiology and pathological mechanism of this disease, no breakthrough has been made in understanding its fundamental pathological mechanism, although researchers in and outside China have dedicated much research to this topic. This disease is prevalent in male adults aged 30–50 years [2]. Because this disease develops rapidly, it can develop to femoral head collapse in 1–4 years in approximately 80% of patients who do not receive surgery during the early stages [3]. Thus, ONFH is a common disease that causes the dysfunction or functional loss of hip joints among young adults [4]. Even in cases of asymptomatic ONFH, the disease will ultimately develop into surface collapse and osteoarthritis in approximately 60% of patients [5], who will require total hip arthroplasty (THA) [6]. THA (THA is a procedure that replaces the hip joint. The joint consists of two parts: the acetabulum and the femoral head. During the operation, these two parts are removed and replaced by artificial prosthesis.) for the treatment of ONFH can not only relieve hip pain but can also improve the function of the hip joints and is currently one of the most successful orthopaedic surgeries. However, complications after THA and the long-term survival rate remain the main problems faced by young patients [7, 8]. Therefore, the preservation of the hip joint function is the primary goal in the treatment of ONFH in young adult patients.

At present, the hip-preserving surgeries commonly performed in clinic mainly included core decompression, non-vascularized bone transplantation, vascularized bone transplantation, tantalum rod implantation and osteotomy [9, 10]. However, the problems to be solved in the different stages of ONFH differ. Core decompression was used earlier in the hip-preserving treatment for ONFH, which remains as the gold standard for the treatment of ARCO stage I ONFH at present [11]. For advanced ONFH, the larger diameter of the decompression hole might destroy the mechanical support structure of the femur head, leading to iatrogenic collapse. Hence, this is generally performed in combination with non-vascularized bone transplantation. The bone tunnel formed by drilling decompression provides a basis for the bone transplantation. The transferred bones could fill up the cavity of the cleared necrotic lesion and temporarily become subchondral supporting structures, promoting new bone regeneration *via* bone induction or bone formation. Therefore, in the early stage of ONFH, the implant materials should have the function of inducing new bone regeneration. The femoral head would gradually collapse with the progression of ONFH. Hence, it is particularly important to provide strong mechanical support at this stage. Porous metal implantation emerged based on the theory of core decompression and non-vascularized bone transplantation. The porous metal is a kind of special material that could induce the regeneration of new bone *via* its characteristic porosity. In clinic, the porous tantalum rod is the most widely used due to its good biocompatibility, similar elastic modulus to human bones and cancellous bone-like honeycomb structure. This could provide mechanical support for the subchondral bone, and benefit bone growth at one time [12]. The appearance of porous tantalum rod provides an ideal bone implant material for the treatment of femoral head necrosis. In order to improve the ischaemia condition in the necrotic femoral head, scholars have attempted to achieve revascularization through vascularized bone transplantation [9, 10, 13–15]. The principle of vascularized bone transplantation in the treatment of ONFH was not only to reduce the pressure and remove the necrotic bone, but also to promote the reconstruction of the subchondral bone *via* the implantation of normal bone and reconstruction of blood circulation, providing osteoinductive cells. However, there were problems of falling off or sinking of the transferred bone flap in clinical use. Hence, this needs to be combined with biomaterials to play the roles of fixation and support, and improve the therapeutic effects. The principle of osteotomy was to convert the weight-bearing area into a non-weight-bearing area by changing the angle of the femoral neck and the force distribution of the femoral head. After the osteotomy, the weight-bearing area of the femoral head was replaced by normal bone and articular cartilage [16]. Osteotomy has been generally considered to be suitable for younger patients, in which the ACRO stage II and III, and necrosis range are less than 30%. However, osteotomy can induce serious operation trauma and the destruction of the blood supply in the femoral head, causing the prognosis to remain uncertain. In addition, osteotomy destroys the normal anatomy of the trochanter major and trochanter minor. This requires the patient to undergo a second operation to remove the internal fixation. If the operation fail, it would be hard to switch to the joint replacement. Hence, osteotomy has been rarely performed in the past decade.

Although guidelines for the diagnosis and treatment of ONFH have been provided by many organizations worldwide [1, 4, 17, 18], no unified standards are available for indications for hip surgeries. In addition, there is no consensus on the treatment timing and the choice of surgical method in clinical practice, resulting in differences in the treatment outcomes of hip surgeries [19–22]. To improve the success rate of hip surgeries and extend the lifetimes of hip joints, various biomaterials have been used for the repair and treatment of the femoral head. However, due to the wide variation in these biomaterials, there is no consensus on the implant standard, resulting in different clinical treatment outcomes. Here, we summarize the application of biomaterials for the repair of ONFH with a focus on hip-preserving surgery.

### Non-structural implant materials

Core decompression can remove necrosis and reduce high pressure in the femoral head, enable revascularization and bone regeneration in the necrotic region and thus alleviate the symptoms, and retard the progression of necrosis and promote lesion repair [2]. However, upon removing and decompressing the femoral head, there is no structural support for the subchondral bone plate, which increases the risk of femoral head collapse and femoral neck fracture [7]. Mont *et al*. [23] performed an analysis of 1206 hip core decompression patients and found that in 36%, the necrosis continued to progress after core decompression alone. Scully *et al*. [20] and Goodman [24] also believes that core decompression alone is not enough to prevent the progression of the disease; the subchondral bone must be strongly supported.

At present, the treatments of ONFH by using autogenous bone, allogeneic bone and artificial bone combined with core decompression are different from each other [8, 25]. In recent years, with the development of biomedical materials, the application of bone repair replacement materials is increasing. Bone graft substitutes can fill bone defects and provide certain mechanical support, strengthen the repair of the defect and promote bone healing. At present, the bone graft materials used in repairing the femoral head are mainly made of synthetic bone materials. Although there are a wide variety of artificial bone materials, there have been few experimental studies and clinical applications for the repair of AVN of the femoral head, which commonly use calcium phosphate synthetic materials.

**Figure 1.**
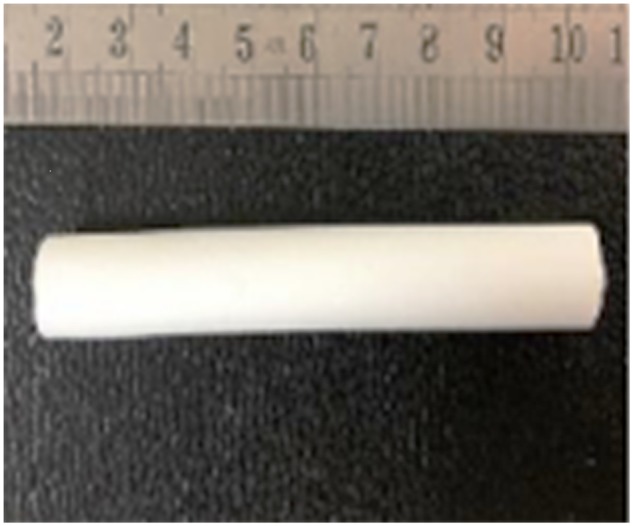
Porous bioceramic rod (diameter: 10 mm, length: 80 mm, macropore: 500–600 μm, interconnection: 120 μm). Reprinted from Ref. [31] with permission

Calcium phosphate (Ca-P) ceramics artificial bone is widely used inorganic bone replacement material, and its main components are calcium and phosphorus, which are very similar to the inorganic components of normal bone tissues. As an ideal scaffold material, Ca-P ceramics not only provide support but also have calcium content similar to those of the human bone tissue mineral, with a Ca/P ratio of less than 1.6; thus, the scaffold has some degradability in the body. Its porous structure provides a three-dimensional (3D) space for the growth of bone cells and the ingrowth of new bone tissues, there by guaranteeing the necessary conditions for bone regeneration and repair [26–28]. Roberto *et al*. [29] used an injectable calcium sulphate/Ca-P bioceramic to treat 37 patients with Steinberg stage Ic–IIIa ONFH. The mean follow-up was 20 months, and the Harris hip scores (HHS) [30] (HHS: The HHS was developed for the assessment the results of hip surgery, and is intended to various hip disabilities and methods of treatment in adult population. The original version was published in 1969.) increased from 68 points pre-operatively to 86 points post-operatively. The radiological results showed that 29 hips (78.4%) improved or had no further collapse. The overall clinical success rate of the procedure was 86.5%. The authors believed that the injection of a bioceramic after core decompression effectively prevented the development of the early-stage collapse of ONFH and even had a certain treatment effect on early-stage collapse. To strengthen the mechanical support function of the bioceramic, some researchers have shaped them into rods (Fig. 1). Lu *et al*. [31] used bioceramic rods (Fig. 1) to treat 72 patients with ARCO stage IIa–IIIc ONFH. The patients had a mean follow-up period of 20 months, and the HHS increased from 58 points pre-operatively to 82 points post-operatively. The overall clinical success rate of the procedure was 90.3%, and the treatment outcome was better than that of patients treated with ceramic bone particles.

Hydroxyapatite with excellent biocompatibility is the most widely studied bioactive material in Ca-P ceramics. The material itself can provide an excellent porous scaffold, newly bone tissues can growth inside the pores and at the interface with the autologous bone. Additionally, chemical bonds form between the scaffold and the contacting bone tissues, which enhance the bonding strength [32–34]. Yamaski *et al*. [35] investigated the transplant of hydroxyapatite to treat ONFH, the results showed that this composite material not only promoted the differentiation and proliferation of bone cells and repaired necrotic bone tissues but also provided mechanical strength, thus effectively preventing the further development of ONFH. Yang *et al*. [36] allocated 64 patients (84 hips) with ONFH (Steinberg stage I, II, and IIIa) into a randomized control clinical trial in which one group was treated with hydroxyapatite and the other group with autologous cancellous bone grafts. Significant differences were found in both the HHS and the Visual Analogue Scale (VAS) [37] outcomes for the two groups of patients pre- and post-surgery, and the hydroxyapatite group performed better than the control group. (VAS is a popular tool for the measurement of pain. It consists of a line usually 100 mm in length, with anchor descriptors. The patient makes a mark reflecting his or her perception, and the distance from the left endpoint to the mark is measured, in mm. The VAS was used for the measurement of pain from the mid-1960s.). Yang believed that the main reason for this was that the mechanical support provided by the hydroxyapatite was stronger than that provided by the cancellous bone.

Although bioceramic materials achieve very good clinical treatment outcomes, they have obvious shortcomings. For example, the degradation time of synthetic materials in the body is difficult to detect and adjust. Some synthetic materials exhibit slow adsorption at filling sites or no adsorption at all, whereas the adsorptions for some synthetic materials at the filling sites are too fast, and as a result, the ideal morphology cannot be maintained. Some artificial bone used as scaffold materials cannot create a sufficient 3D porous structure, which is unfavourable for cell ingrowth. Even worse, some materials experience obvious rejection after implantation into bodies. The bone repair materials that are clinically applied or at the research stage at present are not ideal bone graft substitutes due to their respective defects and challenges [38].

### Implantation of growth factors materials

To overcome the limitations of artificial bone substitute materials that do not enable bone induction and osteogenesis, some scholars have tried to study the composite bone based on growth factors using clinical trials. Both *in vivo* and *in vitro* experiments show that composite materials with growth factors can promote bone growth, collagen synthesis and fracture repair [39]. However, there are only two bone morphogenetic proteins approved in Europe and the USA for clinical application, namely, morphogenetic protein-2 (BMP2) and morphogenetic protein-7 (BMP7) [40].

Sun *et al.* [40] retrospectively compared the clinical outcomes of 72 patients with non-traumatic ONFH (ARCO stage IIb–IIIa) with and without BMP2. Through an average follow-up of 6.1 years, the survival rates for the patient groups treated with and without BMP2 were 81.8% and 71.8%, respectively, and this difference was significant. The authors believe that BMP2 can improve the clinical efficacy and quality of bone repair. However, this study also had limitations. For example, the two types of applied artificial bones were not counted separately. Moreover, the osteogenesis rates of the two types of artificial bones might differ, which could also have influenced the results.

### Structural implantation materials

The application of core decompression combined with non-structural bone-grafting materials achieves excellent clinical outcomes in the treatment of early-stage ONFH. However, due to the lack of effective mechanical support, this method has limited application in collapse period ONFH. Therefore, the search for medical metallic materials that possess excellent mechanical properties is a topic of interest due to their potential to provide effective mechanical support post-femoral head surgery and thus delay or correct femoral head collapse.

#### Application of porous tantalum rods

**Figure 2.**
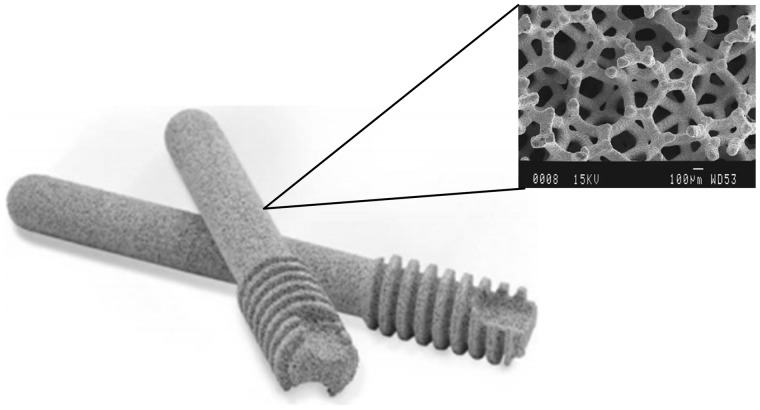
Photograph of the porous tantalum implant. The magnified photograph shows the 3D structure of porous tantalum for scanning electron micrograph. This material has the porosity of 75–85% and the pore size of 400–550 μm

The implantation of porous tantalum rods is a surgical mode derived from the prototype of core decompression. Porous tantalum possesses excellent biocompatibility and mechanical properties. The elastic modulus of porous tantalum lies between that of cancellous bone and cortical bone (Table 1). Porous tantalum has a high porosity and consequently structural and mechanical properties similar to those of human bone tissues (Fig. 2). Implanted porous tantalum rods can support the femoral head, prevent its collapse, and benefit the ingrowth of new vessels and bone tissues to achieve the treatment goal. Therefore, these materials have great potential for ONFH treatment [41, 42].

**Table 1.** Mechanical properties of porous Ta

	Elastic modulus (GPa)	Compression strength (MPa)
Cortical bone	2–30	100
Cancellous bone	0.01–3	2–12
Porous Ta	0.1–30	10–100

In 2005, Tsao *et al*. [43] reported the first multiple-centre clinical study of the application of porous tantalum rods to treat ONFH. One hundred thirteen porous tantalum rods were implanted in 98 ONFH patients, which included 17 Steinberg stage I patients and 96 Steinberg stage II patients. The survival rate of all Steinberg stage II patients was 72.5% at 48 months post-hip surgery, and the HHS increased from 63 to 83. Thus, satisfactory clinical efficacy was achieved. Veillette *et al*. [44] followed up with 52 ONFH patients (58 hips) for 24 months and found that the conventional treatment of early-stage OFHN with core decompression and a porous tantalum implant exhibited advantages over vascularized fibula grafts, such as a short surgery time, little haemorrhaging, a short hospitalization time, no damage to or pain at the bone graft donor site and a short restoration time. ONFH patients with no chronic systemic diseases had an overall survival rate of 92%. Liu *et al*. [45] found that the implantation of tantalum rods for the treatment of Steinberg stage I and II ONFH obtained better clinical results and higher cumulative survival rates than transplantation with traditional composite bone materials. The clinical results from their study showed highly encouraging survival rates and a delay in or prevention of conversion into THA for patients without the use of corticosteroids, especially those with hips without bone marrow oedema. However, the clinical efficacy was not ideal for patients with collapse period ONFH.

With further investigation of the application of tantalum rods for the treatment of ONFH, clinically failed treatments also gradually emerged. Tanzer *et al*. [46] performed histopathology analysis on 15 clinically failed tantalum rod implants and found that osteonecrosis to different extents was present in 14 of the 15 patients, fracture of the subchondral bone of the femoral head was present in all instances, and collapse of the femoral head at 4–11 mm was present in nine instances (60%). Although scanning electron microscopy confirmed the presence of bone ingrowth in 13 (87%) of the 15 patients, most of the newly born bones extended less than 2 mm into the tantalum rods, with a mean extent of 1.9%. Therefore, the bone ingrowth around the tantalum rods was not sufficient. Floerkemeier *et al*. [2] assessed the treatment of 19 ONFH patients (23 hips) with tantalum implants after core decompression, and the follow-up showed that 13 cases needed the THA after the surgery. The survival rate after the implantation of an osteonecrosis intervention rod after a mean follow-up of 529 days was only 44%, and the outcome after core decompression combined with the insertion of a tantalum osteonecrosis intervention implant did not show superior results compared with core decompression alone. Zhang *et al*. [47] analyzed 4 failed cases among 13 patients who received a tantalum rod implant and believed that rather than insufficient mechanical support resulting in the improper positioning of the implants and inadequate bone ingrowth, the nullification of the core decompression and consequential intra-osseous pressurization probably led to the early failure of the porous tantalum implants at the early stages. However, there is no consensus on the failure mechanism of porous tantalum implants for the treatment of ONFH.

#### Application of porous titanium rods

**Figure 3.**
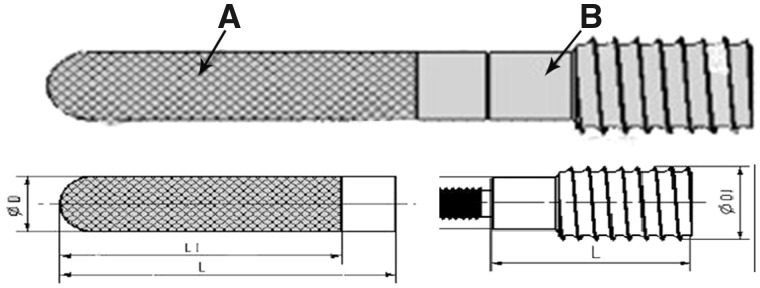
Trabecular metal structure of titanium metal trabecular bone reconstruction system. (**A**) The bone trabecular holder portion. (**B**) The connecting rod. Reprinted from Ref. [48] with permission

Zhang *et al*. [48] found that the removal of tantalum rods from patients with failed ONFH treatment with porous tantalum rods for THA was difficult. The end of the tantalum rods needed to be cut, and then the tail of the tantalum rods was carefully extended with bone cement. The rods were retrieved, the femoral stem prosthesis was installed and sufficient bone grafting was finally performed in the proximity of the prosthesis. There is an increased risk of surgery and postoperative complications. With the development of 3D printing technology, it is possible to fabricate biomaterials with complex architectures [49–54].Titanium alloy has been widely used in clinic due to its good biocompatibility, high corrosion resistance and superior mechanical property [55, 56]. Based on this, some scholars have introduced a 3D pore structure into titanium alloy, and an ideal biomimetic architecture that has both excellent biocompatibility and mechanical properties for load-bearing bone reconstruction was proposed. Besides that, porous titanium would have adjustable elastic modulus by altering its porosity to reduce stress shielding [57–59]. Therefore, porous titanium and titanium alloys for biomedical applications were of great interest in recent years, for example, Zhang *et al*. [60] provide an effective method to build orthopaedic implants with personalized shape and adjustable mechanical properties. Biomimetic architectures with appropriate porosities and mechanical properties allow bone ingrowth and avoid stress shielding. Biomimetic architectures porous Ti6Al4V scaffolds were custom-made with suitable mechanical properties for load-bearing bone tissue reconstruction. Based on the above research, Liu *et al*. prepared a porous titanium trabecular support device using 3D printing (Fig. 3). The support was made based on a split design in which the tail was the connecting rod for easy removal at a later time point. The porosity of the tail end was 50–80%, and the tail end had a compressive strength greater than 20 MPa. As a result, human bone tissue could easily in-grow on the trabecular support device to achieve the integration of the human bone with the device [61].

Zhang *et al*. [48] compared the clinical outcomes of titanium and tantalum rods based on a prospective study. Each group was comprised of 30 ARCO stage II patients, and the patients were followed up at 6, 12 and 24 months post-surgery. At the 6-month follow-up, the HHS was lower for the tantalum group than for the titanium group, but no significant difference was observed between the two groups at the other time points. The improvement was high at IIa and IIb stages post-surgery, although no significant difference was found for the recovery and hip preservation rates. However, as time progressed, ONFH development was noted for both groups of patients, and the number of patients who reached the standard of THA increased, suggesting that surgery to improve the support strength alone could not completely prevent ONFH development.

Although porous titanium rods exhibit no obvious difference in the clinical treatment of early-stage ONFH compared with porous tantalum rods, their porosity and elastic modulus are significantly different. The advantages of porous titanium include easy removal and no requirement for a large-sample, multicentre, and long-term clinical study. However, its long-term effects remain to be discussed.

#### Application of memory alloys

**Figure 4.**
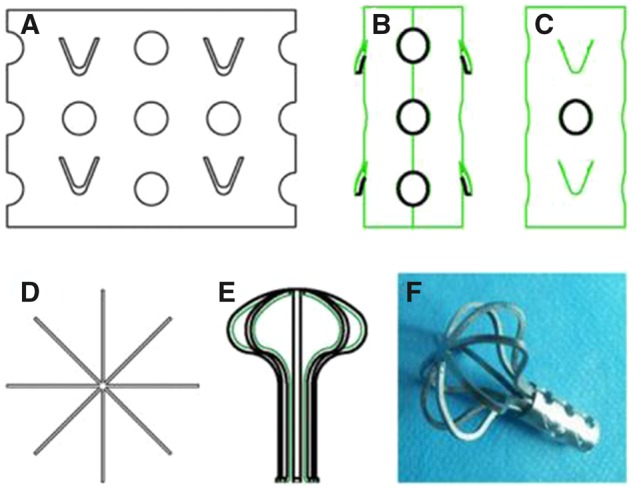
Umbrella-shaped femoral head support device. (**A**) Roughcast of the umbrella frame and sleeve of the femoral head support device. (**B**) Lateral view of the moulded sleeve of the support device. The round hole is the blood supply hole. (**C**) Frontal view of the moulded sleeve of the support device; the V-shaped hole is the non-slip barb hole. (**D**) The umbrella shape of the support device. (**E**) The shape of the moulded umbrella-shaped frame of the femoral head support device. (**F**) The final state of the support device. Reprinted from Ref. [66] with permission

Through the analysis of failed cases of ONFH patients with large necrotic areas who had received porous tantalum rod implants, some researchers believe that tantalum rods with a 10-mm diameter cannot effectively support wide necrotic areas and thus induce a second collapse of the necrotic area [62–64]. To address this problem, Wang *et al*. [65] designed Ni-Ti memory alloy balls to increase the support area. However, during clinical application, the authors found that the supporting force was still small. In addition, the implantation of such support devices requires the opening of the femoral head, which causes large-scale damage. Yu *et al*. [66] produced an umbrella-shaped memory alloy femoral head support device based on the super elasticity and shape memory effect of Ni-Ti memory alloys. An alloy sheet was cut into eight shapes of umbrella ribs that were bent into an umbrella shape to form an umbrella-shaped support device, and support sleeves were joined at the tail ends to form specific memory shapes. Different-sized umbrella-shaped support devices were made to fit different-sized necrotic areas for clearance (Fig. 4), and they were applied to treat 10 ONFH patients (18 hips). According to the Ficat classification, 10 hips were at II stage, 6 hips were at III stage and 2 hips were at IV stage. Except for 1 hip implant that failed and necessitated THA, the remaining 17 hips were followed up with a mean time of 19.7 months. ONFH did not further develop, and the clinical yield was 82.35%. The authors believed that the umbrella-shaped support devices enhanced the mechanical support of the subchondral bones at the weight-bearing area of the femoral head, thereby decreasing the local stress and delaying further development of necrosis. Although the ONFH stage reached Ficat stage III, this method can still be used to treat the disease.

Although medical metal support materials achieve excellent clinical outcomes in the treatment of early-stage ONFH, they also have obvious limitations. For example, support surgery alone cannot prevent ONFH progression. Some researchers believe that the fundamental reason behind this limitation is closely related to the pathological mechanism of ONFH. Artery ischaemia is the ultimate destination of ONFH progression. Support surgery alone cannot fundamentally resolve the problem of ischaemia of the femoral head, which is the key factor that fails to prevent the progression of this disease.

### Vascularized bone grafts

#### Application combined with a magnesium screw

**Figure 5.**
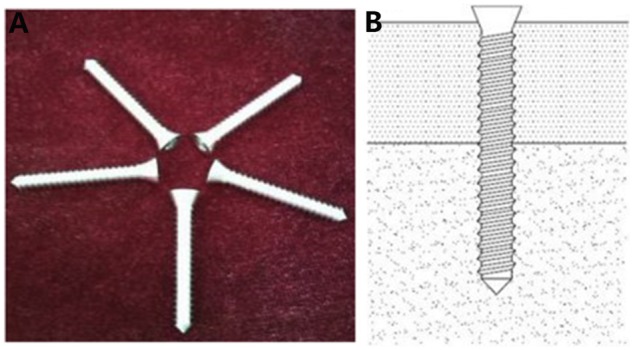
** **Mg-based screws used for fixation of bone flaps. (**A**) Photo of Mg screws (shaft diameter ¼ 4.0 mm and length ¼ 40 mm) with 99.99% purity (4 N). (**B**) Schematic diagram of fixation method with Mg screws. Reprinted from Ref. [73] with permission

**Figure 6.**
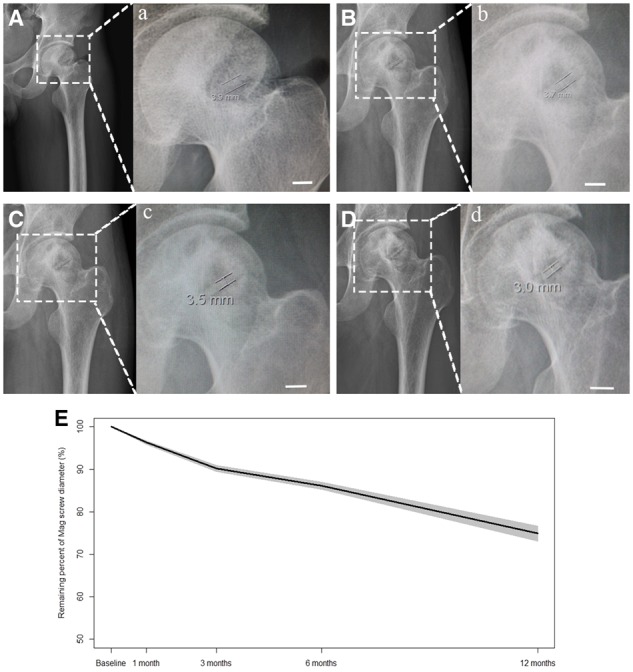
The temporal changes in biodegradation rate of Mg screws interpreted in decrease in screw diameter. (**A**–**D**) X-ray imaging of femoral head in patients performed with Mg screws at 1 (A), 3 (B), 6 (C), and 12 (D) months post-operatively. (**a**–**d**) Magnified surgical regions in (A–D) for measurement of screw diameter at different time points. Scale bar represents 10 mm. (**E**) The remaining percentage in screw diameter over implantation time. The black line stands for means while the grey area represents 95% confidence interval. The average reduction percentage of Mg screw diameter was 3.7%, 9.3%, 13.7%, and 25.2% at 1, 3, 6, and 12 months, posto-peratively. Reprinted from Ref. [73] with permission Figure 6.

Due to the aforementioned problems, some researchers [9, 10, 13, 14] designed vascularized bone-grafting method. This method not only fundamentally resolves the blood supply problem but also utilizes certain mechanical properties of the bone graft. As a result, it can fill the femoral head after clearing the necrotic bone and prevent collapse. This surgical method combines the advantages of both biological and mechanical restoration, thereby expanding the applicability of hip-preserving surgery and improving the clinical efficacy of hip-preserving surgery at the collapse stage. There are even reports of the successful application of this surgical method at the late stage of collapse [67]. However, during follow-up, bone flap displacement and detachment are present in some patients. Therefore, Zhao *et al*. used degradable magnesium materials to fix the implanted bone graft (Fig. 5), prevent the loosening and detachment of the bone graft, and further enhance the hip preservation treatment efficacy. The advantages of the application of degradable magnesium screws for fixation are as follows: (i) the density of magnesium (1.74 g/cm^3^) is very close to the density of human bone (2.1 g/cm^3^) [68–70]; (ii) magnesium possesses excellent biocompatibility, is abundant in human bone, and can promote the generation of new bone and the metabolism of bone tissues [71–73]; (iii) magnesium can be naturally degraded in the human body, and thus no reoperation is required to remove the magnesium screws, which not only lessens the pain of the patients but also reduces their economic burden. Zhao *et al*. [73] performed randomized clinical trials on 48 ONFH patients (ARCO stage II and III). During the 12-month follow-up period after surgery, the control group and the group with magnesium screws for fixation exhibited significantly different extents of necrosis recovery; the bone grafting of the magnesium screw group better fused with the surrounding bone tissues, the hip joint of the magnesium screw group exhibited higher bone growth levels and the HHS of the magnesium screw group was higher than that of the control group. X-ray imaging analysis was used to monitor changes in the diameters of the magnesium screws at different times, and an approximately 25% reduction in the volume of the magnesium screws was observed at 12 months post-surgery (Fig. 6). However, the screw shape did not show significant alterations and still provided its fixation function, thus improving the success rate of the hip-preserving surgery.

#### Application combined with porous tantalum rods

**Figure 7.**
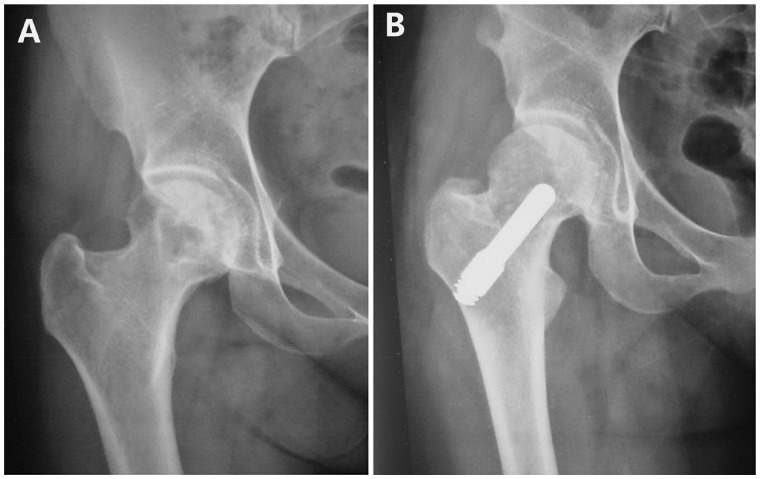
A 38-year-old female with ARCO stage III ONFH (right hip) received the treatment of tantalum rod implantation combined with vascularized iliac grafting. Radiographs were taken pre-operation (**A**), 36 months post-operation (**B**). (B) The joint space remains preserved, the iliac bone graft is well incorporated. Reprinted from Ref. [75] with permission

Zhao *et al*. [74] observed during the clinical follow-up that although the implanted bone graft survived, the femoral head still collapsed in some patients. The main reason for this collapse was that the mechanical support for the implanted bone graft was realized through limited compression, and the support force was very weak. To address this problem, Zhao hypothesized that vascularized bone grafting combined with the implantation of tantalum rods could be used to treat these ONFH patients and take advantages of both approaches. The assured blood supply to the nutrient supply vessels for the vascularized bone graft results in excellent reconstruction of the blood supply to the femoral head. The implantation of tantalum rods not only provides strong mechanical support to the vascularized bone graft but also promotes the generation and repair of bone because it simulates the porous network structure of cancellous bone. Zhao *et al*. [75] reported a technique that combined tantalum rod implantation with vascularized iliac grafting for the treatment of ONFH patients (56 hips) at ARCO stage II, III and IV. All patients were followed up for a mean period of 60 months, and the 5-year joint-preserving success rate of the entire group was 87.5%, with 95% for ARCO stage II, 92% for ARCO stage III and 63.6% for ARCO stage IV (Fig. 7). Patients treated with this combined technique have higher survival rates than those treated with the application of tantalum rods alone. In addition, the hip preservation was expanded to ARCO stage IV patients, further extending the applicability of the hip-preserving treatment.

## Summary and outlook

In summary, ONFH treatment is still a challenge in orthopaedics. THA should not become the main treatment method. In contrast, a successful hip-preserving treatment relies on timing, an accurate diagnosis and a thorough understanding of the pathological changes of ONFH. In the early stage of femoral head necrosis, which is ARCO stage I, the pressure to increase inside the femoral head, which is the main cause of ONFH. Core decompression can effectively relieve the pressure inside the femoral head and shows reliable treatment outcomes at the early stages. For patients with femoral head necrosis developing to ARCO stage II, the extent of necrosis is relatively large, and core decompression alone cannot prevent the collapse of the femoral head. The disease at this stage can be treated with core decompression combined with non-structural bone grafting or metal implantation to provide mechanical support on top of the pressure reduction, prevent the collapse of the femoral head. If the femoral head collapse, the femoral head necrosis progresses to the later period. We recommend that vascularized bone grafting is used in combination with tantalum rod implantation, the assured blood supply to the vascularized bone graft results in excellent reconstruction of the blood supply to the femoral head. The implantation of a tantalum rod can not only provide strong mechanical support to the vascularized bone graft but can also promote the generation and repair of the bone because it simulates the porous network structure of cancellous bone. Consequently, the bone tissue inside the femoral head can rapidly grow into the pores on the tantalum surface and ensure that the bone tissues inside the femoral head and the tantalum rod are strongly bonded to provide long-term stability. Thus, this study offers an effective treatment for late-stage ONFH.

Biomaterials have achieved good therapeutic effects in the repair of ONFH. However, these bone implant materials have certain limitations and have not achieved ideal therapeutic effects. The ideal bone implant material should include biocompatibility, bone induction, bone conduction and porous structure. After being implanted into the human body, it can induce new bone formation, the porous structure provides a 3D space for the growth of the new bone tissue, and the implant material is integrated with the human bone to form a new femoral head. At present, the treatment of ONFH is mostly single material implantation, and the treatment effect is limited. With the development of tissue engineering, it provides a new idea for hip-preserving surgery. If we combine porous materials and growth factors to give full play to the advantages of multiple materials, can we achieve the ideal therapeutic effect? This needs to be tested continuously in clinical practice. We believe that with the development and advancement of material processing technology, and particularly the development of tissue engineering, will certainly improve the treatment outcomes of medical implant products, thereby providing new thinking for hip-preserving treatment. This outcome will improve the success rate of hip-preserving surgery and the quality of life of patients and delay or even avoid the use of THA.
